# Updates on Oxaliplatin-Induced Peripheral Neurotoxicity (OXAIPN)

**DOI:** 10.3390/toxics3020187

**Published:** 2015-05-29

**Authors:** Andreas A. Argyriou

**Affiliations:** Department of Neurology, “Saint Andrew’s” General Hospital of Patras, Patras 26335, Greece; E-Mail: andargyriou@yahoo.gr; Tel.: +302-610-227-907; Fax: +302-610-227-909

**Keywords:** chemotherapy, oxaliplatin, peripheral neurotoxicity, incidence, characteristics, pharmacogenetics

## Abstract

Oxaliplatin-induced peripheral neuropathy (OXAIPN) is of great clinical interest as it ranks among the most common dose limiting toxicities of oxaliplatin (OXA) administration with an obvious impact on the outcome of cancer patients. In addition, OXAIPN has a detrimental effect on the quality of life of cancer patients because it can be long lasting or even permanent. It has a unique spectrum of clinical presentation, being manifested with two distinct syndromes: the acute neurotoxicity that appears soon after OXA administration and is usually transient, and the chronic cumulative syndrome that resembles the characteristics of all platinum compounds. Despite advances in research in relation to the elucidation of the true OXAIPN pathogenesis, characteristics and management, there are still several open issues to be addressed. One of the most important open issues is to determine reliable biomarkers to allow prompt identification of patients at high risk to develop OXAIPN and towards this view well designed genome wide analyses are warranted to adequately address this gap in knowledge. Recent updates are provided in this article in relation to the pathogenesis, clinical characteristics, pharmacogenetics and management of OXAIPN.

## 1. Introduction

Recent advances in the treatment of cancer with antineoplastic agents have significantly increased the survival rates of patients. Among platinum compounds, oxaliplatin (OXA) has demonstrated, in clinical trials but also in everyday clinical practice, its significant activity against colorectal cancer, both in the adjuvant or metastatic setting [1]. Other indications for OXA treatment include rectal, pancreatic and gastric malignancies. OXA acts by blocking DNA replication and transcription, causing cell death and is typically administered with 5-fluorouracil (5-FU) and leucovorin in a combination regimen, known as FOLFOX, or in combination with capecitabine orally administered in a combination regimen, known as XELOX. Particularly, OXA-based chemotherapy with 5FU/Capecitabine backboned chemotherapy seems to provide the optimal impact on progression-free and overall survival [1].

Oxaliplatin-induced peripheral neurotoxicity (OXAIPN), in the form of the acute and chronic clinical syndrome, ranks among the most frequent non-hematological toxicity secondary to the use of OXA-based treatment [2]. It is considered as being the major dose limiting factor, second to hematological toxicities with an obvious impact on the outcome of cancer patients in case of changes in the treatment plan [3]. In addition, it can be detrimental to the quality of life (QOL) of cancer survivors, because it can be long-lasting or even permanent [4]. It is herein provided recent updates in relation to the pathogenesis, clinical characteristics, pharmacogenetics and management of OXAIPN.

## 2. Pathogenesis

The pathogenesis of OXAIPN still remains elusive. However, as concerns the chronic clinical form that closely resembles the characteristics of cisplatin-induced peripheral neurotoxicity [5], it is acknowledged that the dorsal root ganglia (DRG) of the primary sensory neurons are the most common neuronal target of OXA [2]. As a result, there is evidence of DRG apoptosis with degeneration of axons, starting at the periphery and progressing up towards the neuronal cell body in a dying back process [6].

Other, morphologic and functional changes in the DRG cells disturbing normal axonal transport have also been proposed to contribute in OXAIPN genesis. Specifically, mitochondria dysfunction can affect energy balance, and mitochondria flux trough the axon can be disturbed by mitotoxicity or by abnormal axonal transport due to alteration of the cytoskeleton, thoroughly disturbing the normal functioning of the cell. Moreover, DNA damage signaling alters nuclear structure and deregulates protein synthesis [7].

**Figure 1:**
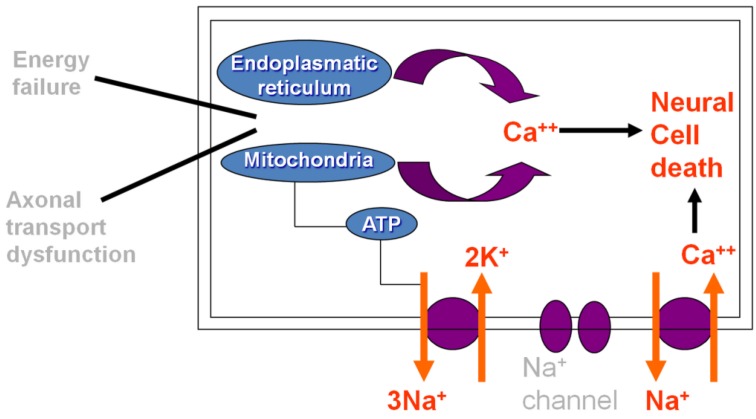
Schematic diagram describing the mechanism of acute oxaliplatin-induced peripheral neuropathy.

On the other hand, it is demonstrated that OXA interacts with ion channels located in the cellular membrane, mostly impairing nodal axonal voltage-gated Na^+^ channels through a pathway involving Ca^2+^ ions. As such, the acute form is thought to be caused by neuronal cell death resulting from dysfunction of axoplasmatic transport and cellular metabolism, but also attributed to the rapid chelation of Ca^2+^ by OXA-induced oxalate [8]. Figure 1 describes the mechanism of acute OXAIPN. 

Nevertheless, apart from peripheral nervous system damage, alterations of the central nervous system (CNS) seem to be also implicated in OXAIPN. At least for the toxicity exacerbated by repeated treatments, damage or response to the CNS are shown and related to pain. The relevance of central sensitization has been preclinically demonstrated also for chemotherapy-induced neuropathies, and further the possibility of anticancer drugs to directly interact and damage the CNS may be not excluded. OXA is able to induce oxidative damage in rat PNS as well as in the spinal cord and it seems that there is a relationship between oxidative stress and neuropathic pain [9]. Moreover, *in vivo* electrophysiological alterations in spinal dorsal horn (SDH) wide dynamic range neurons were described in the spinal cord of OXA-treated mice, thus suggesting that OXA also impacts on the nocifensive behavior and central nervous system excitability to produce neuropathic pain [10]. The central effect is not just focused on neurons but involve also glial cells, as in a recently published experimental study in rats, wherein it was shown that OXA activates both microglia and astrocytes in spinal cord and brain areas [11]. These effects are strongly related with pain since glia inhibitors reduced oxaliplatin-dependent hypersensitivity, thoroughly suggesting that modulation of glial signaling might be an attractive target to treated OXA-related neuropathic pain [12].

## 3. Incidence, Severity and Risk Factors

As to the acute form of OXAIPN, existing knowledge shows that up to 90% of patients treated with OXA-based regimens at a dose ranging from 85 to 130 mg/m^2^ would experience any grade of neurotoxicity. Consequently, a significant enough percentage of about 22% of them would necessitate prolongation of OXA infusion or treatment discontinuation because of treatment emergent acute OXAIPN [13].

Our experience concurs with those data on the incidence and severity of the acute OXAIPN, as in a previously published study by our group in a large homogenous series of colorectal cancer patients we have demonstrated with the use of the Sanofi scale on the oxaliplatin-induced neurotoxicity that the acute form of OXAIPN was present in 146/170 patients (85.9%) treated with either FOLFOX or XELOX, whereas severe acute OXLIPN, requiring prolongation of OXL infusion from 2 to 4–6 h, occurred in 32/146 patients (21.9%) [14]. The main triggers of acute OXAIPN development included the cold temperature because cold exposure is able to further affect sodium channel kinetics, thus predisposing to ectopic activity [15], and the 2 h time of OXA infusion.

On the other hand, available data from older pivotal large studies show the incidence of chronic OXAIPN can be up to 75% in OXA-treated patients [16,17]. Again, recent evidence from our group in a large homogenous series of 200 OXA treated colorectal cancer patients supports the expected incidence rate of chronic OXAIPN. Grading the incidence and severity of cumulative OXAIPN with the use of the clinical version of the Total Neuropathy Score and the neurosensory NCI-CTv3 criteria, we have found that 72.5% of enrolled patients (145/200) had experienced chronic OXAIPN of any grade after OXA-based treatment discontinuation. Clinically significant grade 2 chronic OXAIPN was the most commonly observed severity grade. According to TNSc, the severity of cumulative OXAIPN at the final follow-up was grade 1 in 50/145 patients (34.5%); grade 2 in 59 (40.5%), and grade 3 in 36 cases (25%) [18].

Subsequent results showed that the final neurological outcome of patients after treatment discontinuation can be predicted by clinical and electrophysiological information obtained at mid-treatment. Specifically, multivariate analysis showed that three variables obtained at intermediate follow-up, namely from the clinical point of view the number of acute symptoms (Odds Ratio, OR: 1.9; Confidence Interval, CI 95%: 1.2–3.2; *p* = 0.012) and from the electrophysiological examination the >30% decrease from the baseline value (prior the initiation of chemotherapy) in radial (OR: 41.4; CI 95%: 4.98–343.1; *p* = 0.001) and dorsal sural (OR: 24.96; CI 95%: 2.6–239.4; *p* = 0.005) nerves sensory action potential amplitudes were able to define patients developing high grade OXAIPN at the end of OXA-based treatment. Therefore, it is advisable to monitor the severity of acute OXAIPN and to perform electrophysiological assessment in order to predict and hopefully prevent the induction of severe OXAIPN [19].

Known triggers of chronic OXAIPN include the cumulative OXA dose, the 2 h time of infusion and the existence of peripheral neuropathy prior to the initiation of chemotherapy [20]. Without a doubt, the cumulative dose is the most important triggering factor of chronic OXAIPN genesis [19]. According to available data, high grade chronic OXAIPN is expected to occur in about 10% of patients receiving cumulated doses ranging from 510 to 765 mg/m^2^, while at full OXA dose intensities (higher than 1000 mg/m^2^) the rate of grade 2–3 neurotoxicity peaks to almost 50% of affected patients [16]. Conversely, the chronic toxicity is expected to appear with subsequent accumulation of OXA dose and more specifically at midtreatment, *i.e.*, course 6 for FOLFOX and course 4 for XELOX [2,3].

Moreover, recent evidence shows that the acute OXA effects also represent a trigger of chronic OXAIPN. In a homogenous series of 150 OXA-treated colorectal patients it was evident that those patients with worse acute OXAIPN severities appear to have more eventual chronic peripheral neurotoxicity at the end of treatment due to prolonged activation of voltage-gated sodium channels, induction of cellular stress, and secondary further affectation of sensory nerve cells [14].

Little is known about whether the OXA regimen might also represent a risk factor of chronic OXAIPN and if FOLFOX or XELOX is safer in terms of neurotoxicity. According to previously published retrospective data, XELOX was more potent neurotoxic regimen compared to FOLFOX [21]. Our prospective experience contradicts the latter view, as we have shown that although both regimens demonstrated equally acute neurotoxicity profiles, XELOX may be the preferable regimen to avoid a significant cumulative neurotoxicity associated with FOLFOX, because FOLFOX was associated with increased incidence of chronic neurotoxicity, compared to XELOX-treated patients (*n* = 64/77 *vs.* 44/73; *p* = 0.002), at comparable OXA median cumulative dose that was eventually delivered at the end of chemotherapy for both regimens [22].

Finally, the association of advanced age and increased risk of developing is conflictingly addressed in the literature with reports suggesting that older age is not associated with increased peripheral neurotoxicity, whereas others point otherwise [23,24].

Our experience, in a study sample of 145 OXA-treated colorectal cancer patients, shows that the advanced age does not seem to represent a significant risk factor of OXAIPN in patients with good functional and performance status, compared to younger patients. With a cut-off point of 68 years for defining elderly, there was lack of statistically significant difference in the incidence of both the acute (*n* = 64/75 *vs.* 56/70; *p* = 0.510) and cumulative OXAIPN (*n* = 51/75 *vs.* 49/70; *p* = 0.858) between age groups. The severity of OXAIPN was comparable between age groups [25].

## 4. Clinical and Electrophysiological Characteristics

Distal and perioral cold-induced paresthesias and dysesthesias represent the most commonly reported symptoms of acute OXLIPN and these clinical manifestations appear within hours or days of OXA administration. The repeated exposure and the accumulation of median dose through time with subsequent chemotherapy courses seem to increase both the duration and severity of those hyperexcitability symptoms [26]. However, our experience in 100 OXA-treated colorectal cancer patients shows that other uncommon symptoms which remain unrelated to cold exposure might also be present. A total of 82 patients experienced symptoms of acute OXAIPN, while 45 of them reported uncommon symptoms at significant rates including, shortness of breath, jaw spasm, fasciculations, muscle cramps, and swallowing difficulties [27]. OXA-induces excessive excitability of peripheral motor and sensory nerve fibers as also of cranial nerves, as documented by recording of repetitive compound action potentials, high-frequency discharges of motor unit multiplets and bursts of muscle fiber action potentials, holds response for the clinical manifestation of these uncommon symptoms [28].

Patients having the chronic form of OXAIPN usually complain about distally attenuated paresthesias and dysesthesias in hands and feet in a stocking-and-glove distribution. The element of neuropathic pain in distal extremities is commonly seen. Clinical examination reveals decreased vibration and proprioception and suppression or loss of DTRs in proportion to sensory loss [29]. Motor and autonomic involvement is rarely seen. During electrophysiological assessment there is evidence of decreased or abolished sensory action potentials (aSAP) and normal sensory conduction velocities, in keeping with a sensory neuronopathy. The recording of the aSAP of the dorsal sural nerve together with radial and sural nerves aSAPs appears to be sensitive enough to provide reliable electrophysiological information regarding the chronic form of OXAIPN [19]. Motor nerve conduction study is normal [3]. It should be acknowledged that electrophysiological examination could not be easily performed to every patient because it is a relatively expensive examination as also because it is not always available in the general setting. Towards this view, a recently published study attempted to identify associations between clinical examinations and neurophysiological abnormalities in neurotoxicity-affected cancer patients. It has been shown that abnormalities in vibration and monofilament examinations correspond well to abnormal sural nerve amplitudes and could as such be used into standard practice, where electrophysiology is unavailable, to accurately grade the severity of chemotherapy-induced peripheral neurotoxicity, including OXAIPN [30].

## 5. Long Term Outcome

The chronic form of OXAIPN can be long lasting or even permanent in some cases in proportion to the severity of neurotoxicity at the end of treatment, because of the capacity of platinum compounds to accumulate in DRG for a long time. In some cases, the neurotoxicity continues to progress for a time after the discontinuation of chemotherapy (“coasting” phenomenon) [2,3]. A recently published study from our group aimed to identify a potential reversibility of OXAIPN by following-up its long-term course 2 years after discontinuation of OXA-based chemotherapy. The analysis showed that a total of 73/91 (80%) patients experienced OXAIPN at the end of treatment, while persistence of chronic OXAIPN at least two years after the discontinuation of OXA-based chemotherapy emerged in 61/73 patients (83.5%) and complete resolution in 12 (16.5%) patients. Moreover, it was evident that the severity of OXAIPN significantly improved over time with median TNSc values of 9 (range 2–15) at the end of treatment *vs.* 4 (range 2–12) at 2 years afterwards (*p* < 0.001). Our results suggest that the long term persistence of OXAIPN is common and although improvement is observed, recovery might not be complete [31]. Other studies, applying patients’ reported outcome measures to define the frequency of long term OXAIPN, have reached to the same conclusion, thoroughly demonstrating that the persistence of high grade cumulative OXAIPN over long period after treatment discontinuation might be not that uncommon as previously considered [32]. Re-challenge with OXA-based chemotherapy is generally not advisable and should be omitted although there is some evidence from a small-sized retrospective report that when a rechallenge occurs, the neuropathy is not that severe [33].

## 6. Pharmacogenetics

Only a proportion of OXA-treated patients develop treatment emergent and persistent OXAIPN and to date it has been impossible to detect high risk patients to develop severe (>grade 2) OXAIPN even among patients who are treated with the same OXA-based regimen. As such, candidate gene approaches have been launched in order to identify reliable biomarkers. In the past several single nuclear polymorphisms (SNPs) in genes mainly involved in the pharmacokinetic and phamacodynamic properties have been proposed as being associated with OXAIPN [9]. The literature contains reports suggesting increased susceptibility to OXAIPN with pharmacogenetic variations in genes encoding for drug transporters (*ABCC1* and ABCG1 genes), detoxification enzymes (*MPO*, *GSTA1*, *GSTM1/3*, *GSTP1* and GSTT1), DNA repair mechanisms (*ERCC2*, *XPA*, *XRCC1* and *ERCC1*) and *integrin B3* (an integral cell-surface protein known to participate in cell adhesion and in cell surface-mediated signaling) *Leu33Pro* polymorphism [9,34,35,36]. Further SCNA SNPs, such as the *SCN2A R19K* polymorphism, have been tested and found not related to OXAIPN manifestation [37].

Recent evidence from our group obviated the importance of genes coding for the voltage-gated sodium channels (e.g., *SCN4A*, *SCN9A* and *SCN10A)*. In an adequately powered, prospective cohort of well-characterized OXA-treated colorectal cancer patients, 200 blood samples were genotyped with real-time PCR, using LNA hydrolysis probes or allele specific primers. The main finding to emerge was that the over-dominant model (CT *vs.* CC+TT) of the skeletal muscle *SCN4A*-rs2302237 and the tetrodotoxin-resistant *SCN10A*-rs1263292 polymorphisms have being significantly associated with increased incidence of acute OXAIPN. The CT model of *SCN4A*-rs2302237 was also able to predict the development of cumulative OXAIPN [18].

Other attempts using genome wide analysis (GWAS) have been made in order to identify potential genetic markers for severe OXAIPN. In a Korean study sample of OXA-treated CRC patients, the performed meta**-**analysis combining the discovery and the validation cohorts, revealed a putative association of 9 SNPs in 8 gene loci (TAC1, FOXC1, ITGA1, ACYP2, DLEU7, BTG4, CAMK2N1, FARS2) with chronic OXAIPN [38]. In a subsequent publication applying similar study design in Japanese colorectal cancer patients, it was evident that only one, *i.e.*, FARS2 of these eight previously identified loci was found to be putatively linked with chronic OXAIPN [39].

As such, there was evidence for independent validation of these findings to show if any these SNPs would reach a genome-wide significance level. Our group performed this kind of replication GWAS analysis and found that none of the polymorphisms investigated in both the Korean and Japanese cohorts was found to be associated with grade ≥2 chronic OXAIPN. Our findings altogether suggested a minor role of the SNPs investigated as genetic determinants of chronic OXAIPN. In our opinion, those contradictory results, justify the launch of replication studies with meta-analysis to validate GWAS findings [40].

## 7. Treatment Options

So far, several agents hold promise for their ability to prevent OXAIPN, including acetylcysteine, amifostine, calcium and magnesium infusions, diethyldithiocarbamate, glutathione, Org 2766, venlafaxine, and oxycarbazepine [26]. The largest debate on those candidate neuroprotective agents took place with calcium and magnesium infusions. Back in 2004, the results of an adequately powered retrospective analysis, showing that Ca/Mg infusions might be able to delay cumulative neuropathy, especially in 85 mg/m^2^ oxaliplatin dosage, prompted many oncologists to start implement this intervention in routine clinical practice [41].

However, three years later, the Combined Oxaliplatin Neuropathy Prevention phase III Trial (CONCEPT) was terminated early, because FOLFOX treated patients assigned to receive iv Ca/Mg had significantly decreased response rates, compared to patients treated with FOLFOX alone [42]. Following evidence that this negative effect of Ca/Mg infusions was false, several clinical trials have been launched to eventually test the neuroprotective effect of this intervention [43]. The results of a large phase 3 trial seem to close this issue by demonstrating that Ca/Mg infusions did not substantially improved or prevent OXAIPN [44]. In any case, pooled analysis of available data show that no agent has been demonstrated its neuroprotective capacity against OXAIPN [45]. As such, it is advisable to adhere to the non-pharmacological stop-and-go approach (*i.e.*, intermittent OXA dosing) as described in OPTIMOX1 trial in order to prevent or delay OXAIPN. Briefly, the approach consists of initiation with the dose-intensive FOLFOX7 regimen for six cycles, followed by infusional 5-FU until tumor progression, and then reintroduction of OXA [46].

Since there is no prophylactic treatment against OXAIPN, interventions remain merely symptomatic to alleviate the element of neuropathic pain. Based on results of well-designed randomized controlled trials, only duloxetine at a dose of 60mg per day has been proven effective in diminishing oxaliplatin-associated neuropathic pain [47]. Anticonvulsants, such as pregabalin might also be an option to alleviate neuropathic pain when used with specific caution on the potential side effects [48].

## 8. Conclusions

OXAIPN is one of the major problems that oncologists are asked to cope because it is the most common non-hematological dose limiting toxicities of OXA-based chemotherapy. Due to the OXA’s capacity to accumulate and deposit in DRG for long time and consequent persistence for long term, OXAIPN often has a detrimental effect on the QOL of affected patients.

Despite advances in research in relation to the elucidation of the true OXAIPN pathogenesis, characteristics and management, there are still several open issues for future research to pursue. One of the most important open issues is to identify reliable genetic biomarkers to detect patients at high risk to develop OXAIPN and towards this view well designed genome wide analyses are warranted to adequately address this gap in knowledge. Nonetheless, OXAIPN remains a very challenging area of research in the wide topic of toxic neuropathies.
